# The Non-linear Relationship between Muscle Voluntary Activation Level and Voluntary Force Measured by the Interpolated Twitch Technique

**DOI:** 10.3390/s100100796

**Published:** 2010-01-21

**Authors:** Yi-Ming Huang, Miao-Ju Hsu, Cheng-Hsiang Lin, Shun-Hwa Wei, Ya-Ju Chang

**Affiliations:** 1 Physical Therapy Department and Graduate Institute of Rehabilitation Science, Chang Gung University, 259, Wen-Hwa 1st Rd, Kweishan, Tao-Yuan, Taiwan; E-Mail: imin127@hotmail.com; 2 Department of Physical Therapy, College of Health Science, Kaohsiung Medical University, Kaohsiung, Taiwan; E-Mail: mjhsu@kmu.edu.tw; 3 Department of Rehabilitation, Kaohsiung Medical University Hospital, Kaohsiung, Taiwan; 4 Department of Statistics, Tunghai University, Taichung, Taiwan; E-Mail: linstat@thu.edu.tw; 5 Graduate Institute of Rehabilitation Science and Technology, National Yang Ming University, Taipei, Taiwan; E-Mail: shunhwa@ym.edu.tw

**Keywords:** interpolated twitch technique, voluntary activation, logistic growth model, superimposed electrical stimulation

## Abstract

Interpolated twitch technique (ITT) is a non-invasive method for assessing the completeness of muscle activation in clinical settings. Voluntary activation level (VA), measured by ITT and estimated by a conventional linear model, was reported to have a non-linear relationship with true voluntary contraction force at higher activation levels. The relationship needs to be further clarified for the correct use by clinicians and researchers. This study was to established a modified voluntary activation (modified VA) and define a valid range by fitting a non-linear logistic growth model. Eight healthy male adults participated in this study. Each subject performed three sets of voluntary isometric ankle plantar flexions at 20, 40, 60, 80 and 100% maximal voluntary contraction (MVC) with real-time feedback on a computer screen. A supramaximal electrical stimulation was applied on tibia nerve at rest and during contractions. The estimated VA was calculated for each contraction. The relationship between the estimated VA and the actual voluntary contraction force was fitted by a logistic growth model. The result showed that according to the upper and lower limit points of the logistic curve, the valid range was between the 95.16% and 10.55% MVC. The modified VA estimated by this logistic growth model demonstrated less error than the conventional model. This study provided a transfer function for the voluntary activation level and defined the valid range which would provide useful information in clinical applications.

## 1. Introduction

Interpolated twitch technique (I.T.T.) is a non-invasive method to study human muscle activation. In clinical rehabilitation, this technique is commonly used for assessing the completeness of muscle activation during voluntary contractions, especially for testing whether a muscle is fully active during a maximal voluntary contraction (MVC) [1]. In addition, this methodology has been widely applied in clinical research related to traumatic joint injury [2,3], arthritis [4,5], muscle pain [6,7] and fatigue issues [8,9], and even in assessing the training effect on the adaptations of the central nervous system [10,11].

The technique is executed with a strong electrical stimulation onto a resting and a contracting muscle. By imposing a supramaximal electrical stimulus on a muscle during a submaximal voluntary contraction, the remaining forces can be evoked. The evoked force is generated from those motor units that have not been recruited [12]. With increasing central drive to exert the force to a higher level, the evoked twitch force should become smaller. If no twitch force can be evoked to the MVC, the voluntary activation level (VA) should be 100%. Based on the assumption mentioned above, a negative relationship exists between amplitude of the twitch force and contraction force [12–15]. Thus, the I.T.T. is suggested to be able to estimate the muscle activation level [16,17]. Conventionally, a linear function (see Formula 1 in Methods) has been used to estimate VA. In this function, *Ti* is the twitch force evoked during a contraction at the time of stimulation and the *Tc* is that evoked in the resting muscle.

A potential problem is that the validation of this relationship between VA and true voluntary contraction force has not yet been formally confirmed. The predictive ability of VA to the true voluntary contraction force has only been speculated. The conventional formula, as described previously, for estimating VA was a linear function. One study showed a linear relationship [18] between twitch size or VA and true voluntary contraction force, but other studies reported that the relationship was not linear [17,19–22]. In healthy young and older adults whose motor units could not be fully activated by maximal efforts [23–25], submaximal muscle activation was found during MVC, indicating that the VA might not be reliable as an estimate of the true maximum force [17,21] in maximal efforts. Namely, using the I.T.T. to assess VA, especially near maximal efforts, might cause problems when the conventional formula is used.

For correct use of the I.T.T. and interpretation of the VA without bias, it is important and valuable to develop a new formula to accurately estimate voluntary activation level and to define the appropriate range for clinical use. The purpose of the study was to validate the I.T.T. by finding the transfer function for muscle activation level and determine valid range. The validity of the new developed transfer function was also compared with the conventional function. The conventional linear function is chosen for comparison is due to that this is the most widely used model in clinics and researches.

## 2. Methods

### 2.1. Participant

Eight male adults with no physical disabilities participated in the present study. Their age, height, and weight were 19.2 ± 1.7 yrs, 172 ± 5.36 cm, and 69 ± 8.23 kg, respectively. None of the subjects had any previous history of neuromuscular or skeletal diseases of the lower extremities. Informed consent was obtained from each subject prior to participation in this study.

### 2.2. Experimental Procedure

The plantar flexion torque was measured with a custom-designed ankle torque measurement system [26]. A force transducer (AWU-250, Genisco Technology) implanted in the torque measurement was electronically coupled to a transducer amplifier (Gould Inc, Valley View, OH, USA) with a gain range from 10 to 500 and a frequency response from dc to 1,000 Hz.

The force of the plantar flexor muscles was elicited by electrical stimulation on the tibial nerve using a constant-current stimulator (Digitimer DS7A, Digitimer Ltd, Welwyn Court, UK) with a range of 100 to 400 V and a constant current up to 1,000 mA. The tibial nerve was stimulated in the popliteal fossa with the cathode placed over the tibial nerve and the anode placed over the patella. A stimulus intensity of 120% (duration = 1 ms) was used to elicit the supramaximal twitch. The sampling rate of force signal was set at 100 Hz, which is high enough for force sample and was used by previous researches [27,28].

During testing, the subject sat on a rigid chair and faced the computer screen. The tested foot was firmly fixed on torque measurement system with the ankle joint kept at a neutral position and the knee flexed to 90 degrees. The subject performed three MVCs of soleus and the force trace was displayed on the computer monitor for real-time feedback. When performing MVCs, the subjects were instructed to fully contract the soleus muscle for five seconds. They were given both verbal encouragement and visual feedback on their force to motivate maximal efforts. The 20, 40, 60, 80 and 100% MVCs were calculated and displayed on the computer monitor. The subject then performed voluntary contractions at 20, 40, 60, 80 and 100% MVCs for three sets. Each of the contraction was sustained for five seconds and a rest period of five seconds was provided between two contractions. The electromyography of tibialis anterior was monitored, but not recorded, to ensure there was no co-contraction of this muscle. The supramaximal stimulus was delivered onto resting soleus muscle and during contractions to elicit the control twitch and the interpolated twitch, respectively.

### 2.3. Data Analysis

The amplitudes of the interpolated twitch and the control twitch were analyzed and the voluntary activations were then calculated. The voluntary activation level was calculated as the interpolated twitch (*Ti*) torque relative to the magnitude of the control twitch (*Tc*) [12]. The formula is listed as follows:

(1) VA=1−Ti/Tc

**Figure 1.**
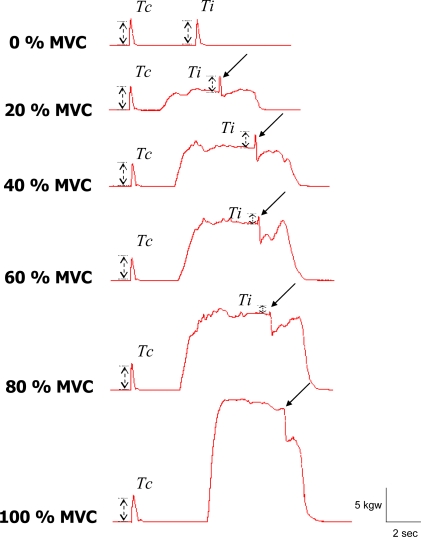
The illustration of I.T.T. at each force level. The *Ti* is the twitch force evoked during a voluntary contracted muscle. The *Tc* is the twitch force that evoked in a resting muscle. Different traces represented the I.T.T. applied on different voluntary force level. While the voluntary forces increased, the superimposed twitch force (*Ti*) decreased (arrows). The steep decline of force after *Ti* was due to the muscle relaxation which was not analyzed in this study.

The *Ti* is the twitch force evoked during a contraction at the time of stimulation and the *Tc* is that evoked in the resting muscle (Figure 1).

In this study, a logistic growth model was used to interpret the curvilinear relationship between true voluntary contraction force and VA. A type of growth form named logistic growth curve is observed to frequently follow an S-shaped or sigmoid pattern when density and time are plotted on arithmetic scales. This model was used not only because it provides non-linear function for sigmoid pattern physiological response but also because the upper and lower limits could be defined for clinical usage in this model. Odum and Barrett [29] pointed out the logistic equation was first proposed by P.F. Verhulst in 1838 and was extensively used by Lotka and “rediscovered” by Pearl and Reed (1920) [30]. Pearl (1925) provided a mathematical derivations and curve-fitting procedures [31]. Logistic functions are good models of biological population growth in species which have grown so large that they are near to saturating their ecosystems.

The equation may be written as follows:

(2) $$\frac{\mathrm{dN}}{\mathrm{dt}}=\mathrm{rN}\frac{\left(K-N\right)}{K}$$

Solving differential Formula (2), we have:

(3) $$N=\frac{K}{1+e^{a-\mathrm{rt}}}$$

where *dN/dt* is the rate of population growth (change in number in time), *r* is the specific growth rate or the maximum possible rate of population growth, *N* is the population size, *K* is the maximum population size possible, or “upper asymptote”, and *a* is the constant of integration defining the position of the curve relative to the origin and is the value of log*_e_*(*K* – *N*)/*N* where *t* = 0.

The Formula (4) for the logistic function can be expressed as follows:

(4) $$y=\frac{C}{1+{\mathrm{Ae}}^{-\mathrm{BX}}}$$

which involves three parameters, *A*, *B* and *C*. The curve starts at *C*/(1 + *A*) when *X* = 0 and increases to an upper limit of *C* when *X* gets large. Assume that the parameters represent positive constants. As the input *X* grows in size, the term *e^−BX^* becomes smaller and smaller. Hence *Ae^−BX^* also becomes smaller and smaller. Therefore, the entire denominator 1 + *Ae^−BX^* is always a number larger than 1 and decreases to 1 as *X* gets larger. Finally then, the value of *y*, which equals *C* divided by this denominator quantity, will always be a number smaller than *C* and increasing to *C*. It follows therefore that the parameter *C* represents the limiting value of the output past which the output cannot grow. On the other hand, when *X* is near 0, the exponential term *Ae^−BX^* in the denominator is a value close to *A* so that the denominator 1 + *Ae^−BX^* is a value near 1 + *A*. Again, since *y* is computed by dividing *C* by this denominator, the value of *y* will be a quantity much smaller than *C*.

To identify the exact meaning of the parameter *A*, set *X* = 0 in the formula; we find that:

(5) $$y\left(0\right)=\frac{C}{1+A}$$

Clearing the denominator gives the equation (1 + *A*)*y*(0) = *C*. Formula (5) means the limiting value *C* is 1 + *A* times the initial output *y*(0). If *B* is positive, the logistic function will always increase, while if *B* is negative, the function will always decrease. The point at which the concavity changes (from concave up to concave down or vice versa) is called the inflection point. For an increasing logistic function, this is the point at which the function is increasing the fastest. For a decreasing logistic function, it is the point at which the function is decreasing the fastest. Thus the inflection point of logistic growth curve is *y* = *C*/2 when X=−ln(1A)/B.

**Figure 2.**
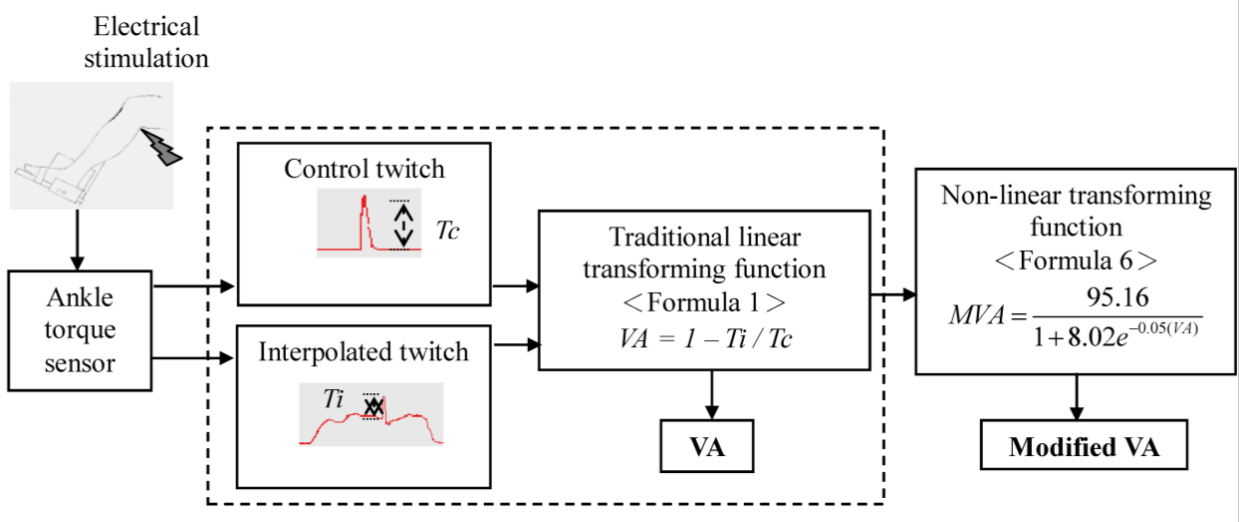
Block diagram illustrating the processing steps of this study. After obtaining *Ti* and *Tc* from I.T.T. technique, VA was obtained from the conventional function. The modified VA was established by fitting the measured to the logistic growth model. The symbol *MVA* refers to the modified VA.

The voluntary activation calculated by this logistic growth model was marked as modified VA in order to be distinguished from the VA which was calculated from the conventional function (Formula 1). The functional block diagram was presented in Figure 2. Descriptive statistics, expressed as Means ± STD, for all eight subjects were used to summarize the results for voluntary activation across force levels.

In order to compare the new model with the conventional model, the difference between the predicted value and the true measured force value were calculated for each model. A paired-t test with p < 0.05 was used to assess if the errors of the two models were different. The root-mean-square errors of both models were also calculated.

## 3. Results

The twitch torque evoked by superimposed electrical stimulation diminished with increasing force levels (Figure 1). The averaged VA across force levels is shown in Table 1. As the force level increased, the voluntary activation increased, however, in a disproportional manner. During 100% MVC, the VA of the subjects ranged from 83.30% to 97.24%, indicating incomplete activation of all subjects.

**Figure 3.**
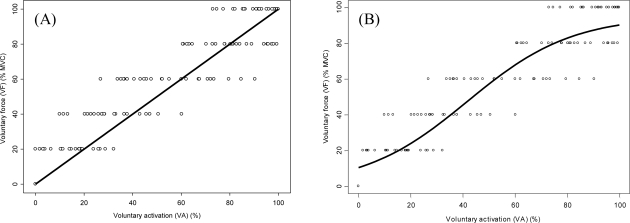
The figure presents the conventional model (A) and the logistic growth model (B) overlaying on the true measured (circles). The conventional model showed a linear relationship between true voluntary contraction force (VF) and the VA. The logistic growth model suggested that VA has a sigmoid relationship with VF. The x axis for both plots is the VA calculated from the conventional model.

The VA and true voluntary contraction force relationship for each individual was somewhat S-shaped or sigmoid (Figure 3). In this study, we fit true voluntary contraction force and VA with a logistic growth model and obtain the following logistic model:

(6) $$\mathrm{MVA}=\frac{95.16}{1+8.02e^{-0.05\left(\mathrm{VA}\right)}}$$

The inflection point of logistic curve is $\mathrm{MVA}=\frac{95.16}{2}=47.58$ at $\mathrm{VA}=\frac{-\text{ln}\left(\frac{1}{8.02}\right)}{0.05}=41.64$.

In Expression (6), the symbol *MVA* refers to the modified VA. The upper limit of the voluntary contraction force is 95.16% MVC and the minimum value is 10.55% MVC when *X* = 0 based on Formula (5). That is, the predictable range of the VA was between the 95.16% and 10.55% of MVC.

For comparing the quality of the modified VA to the VA, the difference between the predicted value and the true voluntary contraction force value were calculated. The results showed that the room-mean-square error calculated from the conventional function (VA) was obviously larger than that calculated from the logistic growth model (modified VA) (15.36% *vs.* 13.49%). The result of paired-t test showed that the absolute errors between two models were significant (*df* = 115, *t* = 2.198, *p* = 0.03). The results suggested that the logistic growth model is better than the conventional model. When the measured points at the high force level that beyond the upper limits predicted by the logistic growth model (>95.16% MVC) were deleted, the root-mean-square error of the conventional model reduced to 14.28%, which were still larger than that calculated from the logistic growth model (13.49%).

## 4. Discussion

The major finding of this study was that voluntary activation level increased by increasing force levels but in a non-linear manner. The logistic growth model produced less error compared to the conventional model. From the obtained upper and lower limit points of the logistic curve, we found the predictable range of the VA and true voluntary contraction force relationship was between the 95.16% and 10.55% of MVC.

The logistic growth model generated errors compared to the conventional linear model, supporting that the relationship between VA and the voluntary force. Previous studies have shown a curvilinear relationship [12,17,19–22]. Belanger and McComas showed non-linearity in the curve of the amplitude of the superimposed twitch against the voluntary force for plantar flexors and tibialis anterior [12]. Very similar to our study, they described the curve as S-shaped with a convex up potion at low contraction intensities and a concave up potion at strong contraction intensities. Bulow *et al.* also demonstrated a curvilinear relationship between superimposed twitch size and true voluntary contraction force for the quadriceps muscles, but the relationship was close to linearity when the force levels greater than 25% MVC [19]. Behm [17] analyzed the model for quadriceps and plantar flexors and reported a plateau of the interpolated twitch ratio (superimposed torque/potentiated torque) and true voluntary contraction force relationship at high contraction intensities, implying that a non-linear relationship existed at near maximal force levels. They also reported the greater linearity was at lower contraction intensities to 60–80% MVC. Stackhouse [22] used a modified method of I.T.T., where the single supramaximal pulse was replaced with a train of supramaximal pulses. A similar term to VA, called central activation ratio (CAR), was calculated and they showed the CAR was insensitive as voluntary efforts greater than 90% MVC. The predictable range of our study was obviously overlapped with those reported in the majority of previous studies [17,19,22]. Most studies, including the present study, indicated that the unpredictable range was located at maximal contraction intensities. We further identified the minimum limit point (10.55% MVC). The force levels below this point were also suggested to be insensitive to be predicted by VA.

This new model is better than the conventional linera one, not only because it generates less error but also because it provides the lower and upper limits for usage. Therefore, the estimated VA will not be negative value or a value greater than 100%. According to the results of this study, if the data beyond the limits were deleted, the performance of the conventional linear model could be improved. Although most of the studies suggested that the conventional model to calculate the voluntary activation might not be valid, the linear model is widely used in clinics because of easy application and non-complicated mathematical transformation. According to our result, establishing new modified VA models for different muscles are suggested while measuring the voluntary activation level on different muscles. If this procedure is not feasible, using only the range between 95.16% and 10.55% in the conventional model is suggested. The underlying mechanism of a VA and true voluntary contraction force curve at low contraction intensities could be related to the contribution of viscoelastic force loss [12,19]. Bülow *et al.* [19] found the resting twitches as well as superimposed twitches at 10% MVC to be smaller than the superimposed twitches on 20–25% MVC and suggested the contribution of viscoelastic force loss. Behm *et al.* [17] further indicated that the additional force loss would happen in the attempt to transfer force through subcutaneous fat and connective tissue. To resolve this problem, Bülow *et al.* [19] suggested a stronger stimulation should be used at low contraction intensities to maintain the validity of the I.T.T. According to Behm *et al.* [17], the non-linear relationship at maximal force levels could attribute to the contribution of synergistic muscles. In our study, plantar flexors muscles, which included the gastrocnemius and soleus muscles, were innervated by the tibial nerve. When the tibial nerve was stimulated, both of the muscles would contract at the same time and contribute to the plantar flexion torque. During testing, we fixed subjects at 90 degrees of knee flexion, which may eliminate the torque of gastrocnemius generated when performing voluntary contraction. The gastrocnemius was considered to have biomechanically disadvantage at this position. Therefore, the contribution of the gastrocnemius was usually ignored while knee flexing to 90 degrees [32,33]. It is possible that as the contraction intensity increased and approached to maximal levels, the disparity of torques would be magnified. The curve therefore appeared non-linear at maximal levels.

Incomplete voluntary activation is a phenomenon that subjects produce submaximal activation during the maximal effort level. Incomplete voluntary activation may also contribute to the non-linearity relation between the VA and true voluntary contraction force. Any twitch force produced above the voluntary maximum is thought to represent incomplete neural drive to the muscle fibers [34]. It has been reported that human subjects can fully activate some of the limb muscles such as adductor pollicis muscle [35] and biceps brachii muscle [1]. Another study reported an inability to fully activate the plantar flexors [12]. In our study, all subjects demonstrated an incomplete activation of the plantar flexors at maximal force level (100% MVC) (Table 1). The failure in full activation could be due to incomplete motor unit recruitment and/or suboptimal motor unit firing rates [24]. Knight [34] established the relationship between motor unit firing rates and incomplete activation. They provided the evidence that lower discharge rates were associated with less activation during maximal isometric contractions.

In addition to the effect of the motor unit firing rates, incomplete motor unit recruitment should be noted. According to the investigation by Kendall *et al.* [36], I.T.T. may highly overestimate the number of motor units activated, since their magnetic resonance imaging (MRI) data showed maximal efforts resulted in the activation of only 75% of muscle fibers of quadriceps. In addition, some studies have also shown that motor unit recruitment is completed by ∼50% MVC in small muscles [37,38] and 70–80% MVC in large muscles [39,40]. For the reasons aforementioned, it is assumed that both the suboptimal discharge rates of recruited motor units and incomplete motor unit recruitment contributed somewhat to the incomplete activation of plantar flexors in the present study.

## 5. Conclusions and Clinical Applications

The modified VA calculated from the logistic growth model significantly reduced the error in comparison to the conventional model while using the interpolated twitch technique. A valid measure range of muscle activation defined by this new model was between 10.55% and 95.16% MVC. A limitation is that the validation is constrained by muscle type, motor unit recruitment and rate coding properties. If this new model establishing process is not feasible in some clinics or researches on other muscles, using only the range between 95.16% and 10.55% in the conventional model is suggested.

The result of the present study provides a valid range of I.T.T. for clinicians to estimate VA, especially for assessing patients who suffer from muscle weakness and could not generate sufficient force with neural inhibition, such as the subjects with joint injury, arthritis and muscle pain. Establishing activation models for other commonly tested muscles and standardizing the technique application procedures and simplifying the calculations by integrating the sensor system on chips are suggested in the future.

## Figures and Tables

**Table 1.** Average and standard deviation of voluntary activation across true voluntary contraction force levels (N = 8).

**Force levels (% of MVC)**	**20**	**40**	**60**	**80**	**100**
Voluntary activation (%)	14.23 ± 7.91	32.05 ± 10.85	54.81 ± 16.04	80.44 ± 11.23	86.71 ± 12.76
